# Modeling the effects of pandemics on physiology-related fertility decline in society

**DOI:** 10.3389/fepid.2026.1740829

**Published:** 2026-09-16

**Authors:** Teddy Lazebnik, Svetlana Bunimovich-Medrazitsky, Ariel Alexi

**Affiliations:** 1 Department of Information Systems, Univeristy of Haifa, Haifa, Israel; 2 Department of Computing, Jonkoping University, Jonkoping, Sweden; 3 Department of Mathematics, Ariel University, Ariel, Israel; 4 Department of Information Science, Bar Ilan University, Ramat Gan, Israel

**Keywords:** demographic fertility, machine learning, pandemic spread, population demographic, SIR model

## Abstract

Pandemics throughout history have raised challenges in many forms, such as increased mortality rates, economic instability, and political shifts. Recent pandemics, coupled with globally declining fertility rates, pose unprecedented demographic challenges for humanity. A pandemic is inherently connected to a population’s fertility in many ways - psychological, economic, and, most importantly, physiological. Nevertheless, this connection is mostly left unintended, blinding us to the effects of the pandemic-caused physiological fertility decline on the population’s growth over time. In this study, we proposed a novel epidemiological model incorporating physiological fertility decline dynamics using an extended Susceptible-Infected-Recovered (SIR) model incorporating age groups, genders, birth and death dynamics, and infection-induced physiological fertility decline. Our model, based on real-world data from 10 large cities globally, demonstrates the impact of infection on fertility, which can persist for generations, creating demographic ripple effects that extend well beyond the immediate pandemic period. That said, the fertility drop is secondary to the pandemic spread, even for relatively high fertility decline due to the pathogen. These results have significant implications for public health policy and population planning. They highlight the need to integrate fertility considerations into pandemic preparedness strategies. Our study provides a quantitative framework for assessing the long-term demographic impacts of pandemics, offering policymakers tools to assess and potentially mitigate the impact of future disease outbreaks at the population level.

## 1 Introduction

Throughout history, pandemics have wielded significant influence on societies spanning centuries, imprinting enduring impacts on mortality rates, economic stability, and political landscapes (1). In recent decades, the persistent specter of emerging and reemerging pandemics and epidemics poses a substantial threat to humanity (2). For instance, the absence of a comprehensive global pandemic agreement has exposed significant deficiencies in managing future outbreaks of infectious diseases (3). While the World Health Organization (WHO) has revised the International Health Regulations (IHR), these updates remain inadequate amidst increasing risks of future pandemics akin to COVID-19 (3). That said, countries are not blind to the potential crisis a large-scale pandemic can bring to their people and prepare on multiple fronts such as healthcare, economics, education, public policy, and international relations (4–8). Despite these efforts, it is currently generally agreed upon that many countries are under-prepared for future pandemics (9).

Such predicted future pandemics will occur in a complex demographic scenario in which fertility is in a constant decline (10). The decline in fertility, which started in the late 18th century and is expected to culminate sometime in the twenty-first century (11, 12), represents a significant demographic shift. In particular, birth rates have dropped significantly during the last decade (13). The continuing drop in fertility rates marks a significant demographic shift driven by multiple societal changes (14, 15). While scholars do not agree if the current trend is positive or negative (16, 17), there is a consensus that an uncontrolled decline in fertility would be devastating for humanity (18).

To this end, a growing body of work is dedicated to the study of factors causing a decrease in fertility. From these studies, several key factors emerged, including changes in social norms (19–22), gender inequality and higher education levels among women (23, 24), and a larger portion of women joining the workforce (25–27). In addition, government policies play a role in influencing the choice to have children (28, 29).

Notably, these factors are all associated with the **decision** to produce fewer offspring rather than the **ability** to produce offspring. However, when we focus on a pandemic, like COVID-19, infection and its long-term outcomes are affecting the ability to produce offspring in addition to the decision to do so (30–34). For example, (35) found that post-COVID-19 effects on female fertility were significant in patients with a history of severe COVID-19 infection. The study revealed a marked decrease in ovarian reserve, hormonal disturbances, and endometrial abnormalities, which are the key factors contributing to infertility. Similarly, (36) discovered that the pathogen at the root of the COVID-19 pandemic adversely affects male fertility more than female fertility, which might lead to fertility challenges after infection. El-Samie et al. (37) showed that SARS-CoV-2 infection can alter ovarian function, disrupt egg development and maturation, lower oocyte quality, and ultimately reduce reproductive function. Hence, an uncontrolled decrease in fertility is an indirect and long-term challenge raised by pandemics (38).

Modeling fertility curves has long interested demographers (39–41). Over the years, various mathematical models have been proposed to describe fertility patterns (42–44). Many of these models have proven to fit age-specific fertility rate distributions of human populations exceptionally well. For example, (42) demonstrated models that accurately represent one-year age-specific fertility rates, and (45) showed how to model both traditional and modern distorted age-specific fertility patterns, fitting a variety of empirical fertility schedules. However, while these models effectively describe fertility, none of them consider the potential impacts of pandemics on fertility trends.

To this end and despite extensive research on the global effects of pandemics on demographic fertility (TFR) decline (46), no mathematical model explicitly captures the connection between pandemic spread and physiological fertility decline dynamics. Thus, in this study, we proposed a novel epidemiological mathematical model that combines the Suscptible-Infected-Recovered (SIR) (47) epidemiological model with physiological fertility decline behavior. Using the proposed model, we investigated *in silico* multiple realistic cases of COVID-19 spread in multiple central cities around the world.

The motivation for the present study stems from a distinction that is largely overlooked in existing pandemic-fertility research. While a substantial body of literature investigates changes in the *decision* to have children during and after a pandemic, an infectious disease may also affect the physiological *ability* to reproduce. Evidence from the COVID-19 pandemic indicates that infection can be associated with alterations in male and female reproductive functions. At the population level, even a moderate physiological reduction in fertility may interact with disease transmission, mortality, aging, and an already declining baseline fertility rate, thereby producing demographic effects that persist after the immediate epidemic has subsided. Nevertheless, conventional epidemic models generally focus on infection, recovery, and mortality and do not explicitly represent this additional physiological pathway. A mathematical framework coupling epidemic dynamics with infection-induced physiological fertility impairment is needed to quantify these long-term demographic effects. As such, the main contribution of this study is a mathematical framework that explicitly couples epidemic and physiological fertility dynamics.

The rest of the paper is organized as follows. Section 2 provides an overview of disease-cased fertility issues and pandemic spread models. Section 3 formally introduces the proposed model. Section 4 presents the experiment design and the obtained results of our simulation-based evaluation using synthetic and real-world data. Finally, in Section 5, we analyze the results and discuss possible future work directions.

## 2 Related work

In this section, we initially provide an overview of disease-cased fertility issues, followed by a review of epidemiological-mathematical models and the agent-based simulation (ABS) method used to solve them numerically.

### 2.1 Disease-cased fertility issues

Fertility is a complex biological process that takes a toll on the woman’s body as it occurs (48). Moreover, the beginning of fertility is also sensitive to multiple physiological properties in both male and female partners (49). As such, it is common to find disease-cased fertility issues from various pathogens (50). For instance, sexually transmitted diseases (STDs), such as HIV, reduce the reproductive capacities of both infected men and women (51–53). However, not only STDs cause infertility. For example, obesity, one of the most common diseases in the US (54), is associated with fertility issues in both genders as multiple studies have highlighted the adverse effects of obesity on both maternal and fetal health during pregnancy, as well as its role in causing infertility (55–59). In a similar manner, (60) show that gynecological cancer treatment significantly affects the fertility of women in reproductive age. This connection is associated with both the effect of the disease itself and the treatments used to cure it such as surgery, chemotherapy, and radiotherapy (61–64).

A special class of disease that causes concern for fertility issues is a viral disease as it spreads quickly in the population and can affect large groups of individuals in a short term of time (65). For instance, Mumps is a repeating endemic worldwide with outbreaks occurring approximately every five years in unvaccinated regions (66). Mumps is a highly contagious and unvaccinated postpubertal male diagnosed with the mumps virus, who frequently develops complications such as mumps orchitis, which often leads to infertility (67). Similarly, COVID-19 in men significantly reduced sperm quality and caused sex hormone disruption, with long-term effects on sperm concentration and total motility. These effects were especially noticeable during the illness stage and can lead to infertility issues in men (68–70). Moreover, (71) showed that the pathogen at the root of the COVID-19 pandemic can affect female fertility and disturb reproductive functions as the virus can infect the ovary, uterus, vagina, and placenta through ACE2, potentially causing infertility, menstrual disorders, and fetal distress.

Despite the immersive impact of epidemics on the population’s fertility, there is no clear model that connects the two on the population level. Thus, in the next section, we review epidemiological mathematical models which can be the basis of a pandemic model that takes into consideration the fertility impact.

### 2.2 Pandemic spread models

Multiple modeling approaches have been used to predict the course of a pandemic (72). One such model is the *SIR* (Susceptible-Infectious-Recovered) model (47), which categorizes the population into susceptible (S), infected (I), and recovered (R) epidemiological states. Namely, susceptible individuals are healthy and susceptible to infection through contact with infected individuals. Infected individuals recover over time at a specified rate and transition into the recovered group, where they gain immunity and cannot be re-infected. Formally, the SIR model takes the following ordinary differential equation representation:

$$\frac{dS(t)}{dt}=-\beta S(t)I(t),\;\frac{dI(t)}{dt}=\beta S(t)I(t)-\gamma I(t),\;\frac{dR(t)}{dt}=\gamma I(t),$$

where β is the average infection rate and γ is the recovery rate.

Although the *SIR* model is often criticized for its simplicity, extensions of this model have demonstrated effectiveness in more complex and realistic situations (73–75). Hence, multiple extensions of the SIR model have been developed to improve the SIR-based models’ expressiveness, prediction accuracy, and robustness (76). One common extension is the SEIR model, which adds an exposed (E) compartment to account for the incubation period of diseases (77, 78).

In addition, various extensions have been proposed to enhance the SIR model and make it more applicable to real-world scenarios. These extensions can be roughly divided into population-related and disease-related. For the population-related extensions, one such extension incorporates birth and death rates, allowing the model to account for the natural increase of the population over time (79). Following this line, age in the form of age groups has been integrated into SIR as different age groups have different epidemiological dynamics (80). For instance, (81) incorporated age-stratified contact information into the temporal compartment SIR model to account for contact patterns of different age groups. These extensions, including birth and death rates, gender disparities, the exposed state, symptomatic and asymptomatic classifications, and age groups, make the SIR model more robust and capable of addressing the complexities of infectious disease transmission. Another important extension considers gender differences (82–84). For instance, (85) proposed a mathematical model describing the transmission dynamics of influenza A viruses, incorporating differences in susceptibility, recovery rates, and vaccination effectiveness between males and females. Accounting for gender is crucial as it influences these factors, which are essential in controlling and eliminating influenza A infections.

For the disease-related extensions, multiple studies introduced the exposed (E) epidemiological state between the susceptible and infected epidemiological states where susceptible individuals that become infected are transformed to the exposed state where they are already infected but not yet infectious which transforms to the infected state, where they also infections, after some period of time (86, 87). For instance, (88) applied a modified SEIR (E - exposed) model, incorporating infection during the latent period and varying levels of population isolation, to predict the spread of COVID-19 and evaluate the effectiveness of different containment measures across 17 regions. Their findings highlighted the need for rapid and drastic isolation to significantly reduce infection rates and fatalities. On top of that, the symptomatic and asymptomatic nature of several pathogens extends the SIR model to include both states (89). For example, (90) extended the SIR model by classifying susceptible individuals as either asymptomatic or symptomatic after exposure, further transitioning them to the infected group based on their symptoms. The authors show this separation allows the model to more accurately capture real-world dynamics, in general, and under different pandemic intervention policies, in particular.

As extended SIR-based models grow in size due to the adoption of the increased number of extensions, such as the ones presented above, ordinary differential equation formulation becomes complex and numerically hard to solve (91). To this end, studies adopted the Agent-based simulations (ABS) approach. Agent-based simulations are preferable over partial or ordinary differential solvers when considering a highly realistic pandemic model or when focusing on a small population size (92–94). These simulations leverage a (extended) SIR model, which tracks the number of individuals in each epidemiological state over time and simulates their interactions to match the overall SIR dynamics. In particular, ABS is used in epidemiological models to address and solve heterogeneous population dynamics, as other methods such as differential equations or functional models are limited in their ability to efficiently describe such dynamics (91). ABS is a computational method used to describe the dynamics resulting from the interactions of various agents, enhancing pandemic models by capturing individual interactions within populations. For example, ABS has been utilized to model COVID-19 spread and evaluate intervention strategies, illustrating how individual behavior influences disease dynamics and the effectiveness of various control measures (90). For instance, (95) tested the inspection unit as a pandemic intervention using an ABS on an extended spatio-temporal SIR to explore a check-point pandemic intervention policy where the agents (i.e., individuals) make heterogeneous decisions about where they are located.

To obtain a realistic pandemic spread model and solve it, we adopted these extensions of the SIR model as well as the ABS approach to solve it for our model.

## 3 Model definition

For the proposed model, we assume a population divided into two genders (male and female) and three age groups (children, adults, and elderly). The children grow into adults after $1/\alpha_{ca}$ years and adults become elderly after additional $1/\alpha_{ae}$ years. The elderly keep living additional $1/\alpha_{ed}(t)$ years until they naturally die from old age. The adult sub-population is fertile and can produce offspring with respect to its fertility status and average reproduction rate (ω). For clarity, we distinguish between two fertility-related quantities. First, π∈[0,1] denotes the probability that an adult who recovers from symptomatic infection develops physiological fertility impairment. Second, r∈[0,1] denotes the proportional reduction in fertility among physiologically impaired adults, and κ:=1−r denotes their residual fertility level relative to adults without impairment. Thus, κ=1 indicates no fertility reduction, whereas smaller values of κ indicate stronger physiological fertility impairment. For simplicity, we assume two fertility states: normal fertility and reduced fertility.

For these settings, let us consider the occurrence of a single-pathogen air-borne pandemic. Each sub-population, defined by its gender and age group, follows fixed possible epidemiological states-susceptible (S), exposed (E), asymptomatic infectious ($I^{a}$), symptomatic infectious ($I^{s}$), and recovered (R). Susceptible individuals can be infected with the disease upon interaction with infectious individuals and transform into the exposed (E) state. Individuals transform to either the asymptomatic infectious ($I^{a}$) or the symptomatic infectious ($I^{s}$) state with probabilities ρ and 1−ρ, respectively, in a rate ϕ of 1/days. Asymptomatic infectious individuals recover at a rate $\gamma^{a}$ 1/days and transform to the recovered (R) state. Symptomatic infectious individuals either recover at a rate $\gamma^{s}$ 1/days and transform to the recovered (R) state or die with probability λ and 1−λ, respectively. Recovered individuals lose their immunity over time and transform back into the susceptible state (S) at a rate δ 1/days. Notably, adult individuals who recover from the symptomatic infectious state develop physiological fertility impairment with probability π. Individuals in the fertility-impaired adult compartment reproduce at a residual fertility level κ=1−r, where r is the proportional fertility reduction caused by the pathogen. Figure 1 presents a schematic view of the proposed model, where panel (A) shows the transformation between epidemiological states for a single gender and sex group, and panel (B) shows the transformation between demographic states and their influence on the population size through birth and death.

**Figure 1:**
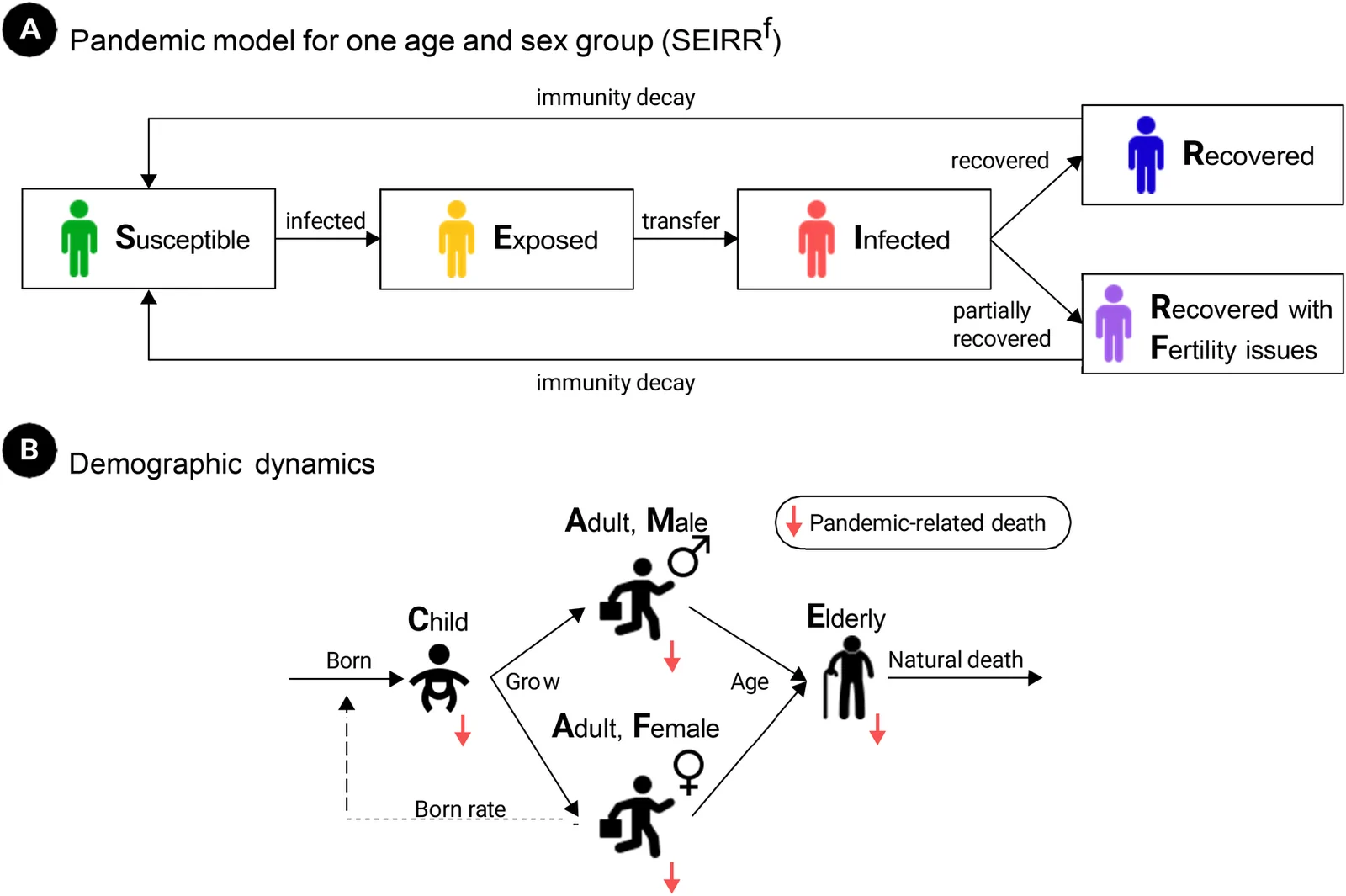
A schematic view of the proposed model. Panel **(A)** shows the transformation between epidemiological states for a single gender and sex group. Panel **(B)** shows the transformation between demographic states and their influence on the population size through born and death.

In order to model the dynamics of the population based on the described scenario, we use a system of ordinary differential equations to present the changes in the various epidemiological states within each sub-population. The model takes the following form: Children (c).

$$\begin{array}{cc} \frac{dS_{g,c}}{dt} & =\tau_{g}\;\mathrm{born}(t)-\mathrm{infect}(S_{g,c}(t))+\delta_{g,c}R_{g,c}(t)-d_{g,c}S_{g,c}(t)-\alpha_{ca}S_{g,c}(t),\\ \frac{dE_{g,c}}{dt} & \;\;\;\;\;\;\;\;\;\;\;\;\;\;\;\;\;\;\;\;\;\;\;\;\;\;\;\;\;\;\;\;\;\;\;\;\;\;\;\;\;\;\;\;\;\;\;\;\;\;\;\;=\mathrm{infect}(S_{g,c}(t))-(\phi_{g,c}+d_{g,c}+\alpha_{ca})E_{g,c}(t),\\ \frac{dI_{g,c}^{a}}{dt} & \;\;\;\;\;\;\;\;\;\;\;\;\;\;\;\;\;\;\;\;\;\;\;\;\;\;\;\;\;\;\;\;\;\;\;\;\;\;\;\;\;\;\;\;\;\;\;\;\;\;\;\;\;\;\;\;=\rho_{g,c}\phi_{g,c}E_{g,c}(t)-(\gamma_{g,c}^{a}+d_{g,c}+\alpha_{ca})I_{g,c}^{a}(t),\\ \frac{dI_{g,c}^{s}}{dt} & \;\;\;\;\;\;\;\;\;\;\;\;\;\;\;\;\;\;\;\;\;\;\;\;\;\;\;\;\;\;\;\;\;\;\;\;\;\;\;\;\;\;=(1-\rho_{g,c})\phi_{g,c}E_{g,c}(t)-(\gamma_{g,c}^{s}+d_{g,c}+\alpha_{ca})I_{g,c}^{s}(t),\\ \frac{dR_{g,c}}{dt} & \;\;\;\;\;\;\;\;\;\;\;\;\;\;\;\;\;\;\;\;\;\;\;\;\;\;\;\;\;\;\;\;=\gamma_{g,c}^{a}I_{g,c}^{a}(t)+\lambda_{g,c}\gamma_{g,c}^{s}I_{g,c}^{s}(t)-(\delta_{g,c}+d_{g,c}+\alpha_{ca})R_{g,c}(t). \end{array}$$ (2a)

Adults without physiological fertility impairment (a).

$$\begin{array}{cc} \frac{dS_{g,a}}{dt} & \;\;\;\;\;\;\;\;\;\;\;\;\;\;\;\;\;\;\;\;\;\;\;\;\;\;\;\;\;\;\;\;\;\;=\alpha_{ca}S_{g,c}(t)-\mathrm{infect}(S_{g,a}(t))+\delta_{g,a}R_{g,a}(t)-(d_{g,a}+\alpha_{ae})S_{g,a}(t),\\ \frac{dE_{g,a}}{dt} & \;\;\;\;\;\;\;\;\;\;\;\;\;\;\;\;\;\;\;\;\;\;\;\;\;\;\;\;\;\;\;\;\;\;\;\;\;\;\;\;\;\;\;\;\;\;\;=\alpha_{ca}E_{g,c}(t)+\mathrm{infect}(S_{g,a}(t))-(\phi_{g,a}+d_{g,a}+\alpha_{ae})E_{g,a}(t),\\ \frac{dI_{g,a}^{a}}{dt} & \;\;\;\;\;\;\;\;\;\;\;\;\;\;\;\;\;\;\;\;\;\;\;\;\;\;\;\;\;\;\;\;\;\;\;\;\;\;\;\;\;\;\;\;\;\;\;\;\;\;=\alpha_{ca}I_{g,c}^{a}(t)+\rho_{g,a}\phi_{g,a}E_{g,a}(t)-(\gamma_{g,a}^{a}+d_{g,a}+\alpha_{ae})I_{g,a}^{a}(t),\\ \frac{dI_{g,a}^{s}}{dt} & \;\;\;\;\;\;\;\;\;\;\;\;\;\;\;\;\;\;\;\;\;\;\;\;\;\;\;\;\;\;\;\;\;\;\;\;\;\;=\alpha_{ca}I_{g,c}^{s}(t)+(1-\rho_{g,a})\phi_{g,a}E_{g,a}(t)-(\gamma_{g,a}^{s}+d_{g,a}+\alpha_{ae})I_{g,a}^{s}(t),\\ \frac{dR_{g,a}}{dt} & =\alpha_{ca}R_{g,c}(t)+\gamma_{g,a}^{a}I_{g,a}^{a}(t)+(1-\pi_{g,a})\lambda_{g,a}\gamma_{g,a}^{s}I_{g,a}^{s}(t)-(\delta_{g,a}+d_{g,a}+\alpha_{ae})R_{g,a}(t). \end{array}$$ (2b)

Adults with physiological fertility impairment (a,f).

$$\begin{array}{cc} \frac{dS_{g,a}^{f}}{dt} & \;\;\;\;\;\;\;\;\;\;\;\;\;\;\;\;\;\;\;\;\;\;\;\;\;\;\;\;\;\;\;\;\;\;\;\;\;\;\;\;\;\;\;\;\;\;\;\;\;\;\;\;\;\;\;\;=\delta_{g,a}R_{g,a}^{f}(t)-\mathrm{infect}(S_{g,a}^{f}(t))-(d_{g,a}+\alpha_{ae})S_{g,a}^{f}(t),\\ \frac{dE_{g,a}^{f}}{dt} & \;\;\;\;\;\;\;\;\;\;\;\;\;\;\;\;\;\;\;\;\;\;\;\;\;\;\;\;\;\;\;\;\;\;\;\;\;\;\;\;\;\;\;\;\;\;\;\;\;\;\;\;\;\;\;\;\;\;\;\;\;\;\;\;\;\;\;\;=\mathrm{infect}(S_{g,a}^{f}(t))-(\phi_{g,a}+d_{g,a}+\alpha_{ae})E_{g,a}^{f}(t),\\ \frac{dI_{g,a}^{a,f}}{dt} & \;\;\;\;\;\;\;\;\;\;\;\;\;\;\;\;\;\;\;\;\;\;\;\;\;\;\;\;\;\;\;\;\;\;\;\;\;\;\;\;\;\;\;\;\;\;\;\;\;\;\;\;\;\;\;\;\;\;\;\;\;\;\;\;\;\;\;\;=\rho_{g,a}\phi_{g,a}E_{g,a}^{f}(t)-(\gamma_{g,a}^{a}+d_{g,a}+\alpha_{ae})I_{g,a}^{a,f}(t),\\ \frac{dI_{g,a}^{s,f}}{dt} & \;\;\;\;\;\;\;\;\;\;\;\;\;\;\;\;\;\;\;\;\;\;\;\;\;\;\;\;\;\;\;\;\;\;\;\;\;\;\;\;\;\;\;\;\;\;\;\;\;\;\;\;\;\;\;\;=(1-\rho_{g,a})\phi_{g,a}E_{g,a}^{f}(t)-(\gamma_{g,a}^{s}+d_{g,a}+\alpha_{ae})I_{g,a}^{s,f}(t),\\ \frac{dR_{g,a}^{f}}{dt} & =\gamma_{g,a}^{a}I_{g,a}^{a,f}(t)+\lambda_{g,a}\gamma_{g,a}^{s}I_{g,a}^{s,f}(t)+\pi_{g,a}\lambda_{g,a}\gamma_{g,a}^{s}I_{g,a}^{s}(t)-(\delta_{g,a}+d_{g,a}+\alpha_{ae})R_{g,a}^{f}(t). \end{array}$$ (2c)

Elderly (e).

$$\begin{array}{cc} \frac{dS_{g,e}}{dt} & \;\;\;\;\;\;\;\;\;\;\;\;\;=\alpha_{ae}\left(S_{g,a}(t)+S_{g,a}^{f}(t)\right)-\mathrm{infect}(S_{g,e}(t))+\delta_{g,e}R_{g,e}(t)-(d_{g,e}+\alpha_{ed})S_{g,e}(t),\\ \frac{dE_{g,e}}{dt} & \;\;\;\;\;\;\;\;\;\;\;\;\;\;\;\;\;\;\;\;\;\;\;\;=\alpha_{ae}\left(E_{g,a}(t)+E_{g,a}^{f}(t)\right)+\mathrm{infect}(S_{g,e}(t))-(\phi_{g,e}+d_{g,e}+\alpha_{ed})E_{g,e}(t),\\ \frac{dI_{g,e}^{a}}{dt} & \;\;\;\;\;\;\;\;\;\;\;\;\;\;\;\;\;\;\;\;\;\;\;\;\;\;\;\;=\alpha_{ae}\left(I_{g,a}^{a}(t)+I_{g,a}^{a,f}(t)\right)+\rho_{g,e}\phi_{g,e}E_{g,e}(t)-(\gamma_{g,e}^{a}+d_{g,e}+\alpha_{ed})I_{g,e}^{a}(t),\\ \frac{dI_{g,e}^{s}}{dt} & \;\;\;\;\;\;\;\;\;\;\;\;\;\;\;=\alpha_{ae}\left(I_{g,a}^{s}(t)+I_{g,a}^{s,f}(t)\right)+(1-\rho_{g,e})\phi_{g,e}E_{g,e}(t)-(\gamma_{g,e}^{s}+d_{g,e}+\alpha_{ed})I_{g,e}^{s}(t),\\ \frac{dR_{g,e}}{dt} & =\alpha_{ae}\left(R_{g,a}(t)+R_{g,a}^{f}(t)\right)+\gamma_{g,e}^{a}I_{g,e}^{a}(t)+\lambda_{g,e}\gamma_{g,e}^{s}I_{g,e}^{s}(t)-(\delta_{g,e}+d_{g,e}+\alpha_{ed})R_{g,e}(t). \end{array}$$ (2d)

where each state described by the gender (g∈{male,female}), age group ({c,a,e}), and

$$\mathrm{infect}(X)\;:\;=\frac{X}{S_{\mathrm{tot}}(t)}\sum_{g\in \{m,f\}}\left[\sum_{i\in \{c,a,e\}}\left(\beta_{g,i}^{s}I_{g,i}^{s}+\beta_{g,i}^{a}I_{g,i}^{a}\right)+\beta_{g,a}^{s,f}I_{g,a}^{s,f}+\beta_{g,a}^{a,f}I_{g,a}^{a,f}\right],$$

where

$$S_{\mathrm{tot}}(t)\;:\;=\sum_{g\in \{m,f\}}\left[\sum_{i\in \{c,a,e\}}S_{g,i}(t)+S_{g,a}^{\;f}(t)\right].$$

Here, $S_{\mathrm{tot}}(t)$ denotes the total susceptible population. This normalization ensures that β retains units of inverse time and that the infection term is consistent across populations of different absolute sizes. The parameter τ∈(0,1) denotes the probability that a newborn is male, and we define

$$\tau_{g}\;:\;=\left\{ \begin{array}{cc} \tau ,\; & g=m,\;\\ 1-\tau ,\; & g=f.\; \end{array}\right.$$

Importantly, epidemiological states ($S,E,I^{a},I^{s},$ and R) that marked by superscript f ($X^{f}$) indicate adults with physiologically fertility-impaired. The birth function depends on the adult population’s gender distribution and fertility status and is defined as follows:

$$\mathrm{births}(t)\;:\;=\frac{\omega }{S(A)}\left(\kappa^{2}A_{m}^{f}A_{f}^{f}+\kappa A_{m}^{f}A_{f}+\kappa A_{m}A_{f}^{f}+A_{m}A_{f}\right)\cdot \min\left(A_{m}^{f}+A_{m},\;A_{f}^{f}+A_{f}\right),$$ (3)

such that $A_{g}\;:\;=S_{g,a}+E_{g,a}+I_{g,a}^{a}+I_{g,a}^{s}+R_{g,a}$ and $A_{g}^{f}\;:\;=S_{g,a}^{f}+E_{g,a}^{f}+I_{g,a}^{a,f}+I_{g,a}^{s,f}+R_{g,a}^{f}$, where g∈{m,f}. The normalization term is defined as

$$S(A)\;:\;=(A_{m}^{f}+A_{m})(A_{f}^{f}+A_{f}).$$

The first factor in Equation 3 represents the average fertility potential of adult male-female pairings, where fertility-impaired individuals reproduce at a fraction κ=1−r of the fertility of unimpaired individuals. The final minimum term limits births by the smaller adult gender group. Here, $A_{g}^{x}$ is the total adult population of gender g∈{m,f} and fertility status x∈{∅,f}. Notably, one could combine $d_{g,e}$ and $\alpha_{ed}$ as both capture the elderly subpopulation decline due to reasons unrelated to the pandemic. However, we pick to keep them separated in this model for formality and keeping the last five equations’ structure as of the other equations. Importantly, we denote by τ∈(0,1) the probability a newborn is male (sex ratio at birth), so that a fraction τ of the total birth flow enters the male child class and a fraction 1−τ enters the female child class.

### 3.1 Mathematical properties of the model

In this section, we establish several qualitative properties of the proposed model, including non-negativity, existence and uniqueness of its solutions, finite-time boundedness, the disease-free state, the epidemic reproduction threshold, and the stability of the disease-free epidemiological dynamics. Formally, let $x(t)\in R_{+}^{n}$ denote the vector containing all epidemiological, demographic, and fertility-related compartments appearing in Equation 2. For convenience, define the total adult male and female populations as $M_{a}(t)\;:\;=A_{m}(t)+A_{m}^{f}(t)$ and $F_{a}(t)\;:\;=A_{f}(t)+A_{f}^{f}(t)$. The birth function in Equation 3 is defined whenever $M_{a}(t)F_{a}(t)>0$. For completeness, at the demographic boundary we adopt the natural convention births(t)=0 if $M_{a}(t)=0$ or $F_{a}(t)=0$. This convention is consistent with the biological interpretation of the model because reproduction cannot occur in the absence of either adult gender.

Theorem 1 **Existence and uniqueness**

Assume that all model parameters are non-negative, that $x(0)=x_{0}\in R_{+}^{n}$ and that $M_{a}(0)>0\wedge F_{a}(0)>0$. Then the model defined by Equation 2 admits a unique solution for every finite time interval.

Proof. The model can be written in vector form as $\frac{dx}{dt}=F(x)$ and $x(0)=x_{0}$. All epidemiological transition terms are linear or bilinear functions of the state variables. Moreover, on the biologically feasible region $\Omega =\{x\in R_{+}^{n}\;:\;M_{a}>0,\;F_{a}>0\}$, the birth function is locally Lipschitz because its denominator is strictly positive and the minimum operator is Lipschitz continuous. Consequently, the complete vector field F is locally Lipschitz on Ω. The Picard–Lindelöf theorem therefore guarantees a unique local solution. Furthermore, the adult populations can leave their respective demographic classes only through finite-rate mortality and aging processes. Hence, for suitable finite constants $C_{m},C_{f}>0$, $\frac{dM_{a}}{dt}\geq -C_{m}M_{a}$ and $\frac{dF_{a}}{dt}\geq -C_{f}F_{a}$. It follows that $M_{a}(t)\geq M_{a}(0)\;e^{-C_{m}t}>0$ and therefore $F_{a}(t)\geq F_{a}(0)\;e^{-C_{f}t}>0$, for every finite t. Therefore, the denominator of the birth function cannot become singular in finite time. Together with the boundedness result established below, this extends the unique solution to every finite time horizon.

Theorem 2 **Positivity**

For any biologically admissible initial condition $x_{0}\in R_{+}^{n}$, all state variables of the model remain non-negative for all t≥0.

Proof. The vector field of Equation 2 is quasi-positive. Namely, when any state variable is equal to zero, all negative terms proportional to that state variable vanish, while the remaining inflow terms are non-negative. For example, at $S_{g,c}=0$,

$${\frac{dS_{g,c}}{dt}|}_{S_{g,c}=0}=\tau_{g}\;\mathrm{births}(t)+\delta_{g,c}R_{g,c}(t)\geq 0,$$

and at $E_{g,c}=0$,

$${\frac{dE_{g,c}}{dt}|}_{E_{g,c}=0}=\mathrm{infect}(S_{g,c}(t))\geq 0.$$

The same property holds for all remaining susceptible, exposed, infectious, recovered, and fertility-impaired compartments. Consequently, no trajectory starting in the non-negative orthant can cross its boundary into the negative orthant, and $x(t)\in R_{+}^{n}$, ∀t≥0.

Theorem 3 **Finite-time boundedness**

Let N(t) denote the total population obtained by summing all compartments of Equation 2. Then $N(t)\leq N(0)\;e^{\omega t/2}$ for every finite t≥0. In particular, the model does not exhibit finite-time blow-up.

Proof. Summing all equations in Equation 2 causes epidemiological transitions, immunity loss, fertility-status transitions, and transitions between adjacent age groups to cancel. Therefore $\frac{dN}{dt}=\mathrm{births}(t)-D(t)$, where D(t)≥0 collects background mortality, old-age mortality, and disease-associated mortality. Because 0≤κ≤1, the fertility-weighted factor appearing in Equation 3 is at most one. Consequently, $\mathrm{births}(t)\leq \omega \min(M_{a}(t),F_{a}(t))$. Since both $M_{a}(t)$ and $F_{a}(t)$ are sub-populations of N(t), $\min(M_{a}(t),F_{a}(t))\leq \frac{N(t)}{2}$, and therefore $\frac{dN}{dt}\leq \frac{\omega }{2}N(t)$. Application of Grönwall’s inequality gives $N(t)\leq N(0)\exp\left(\frac{\omega }{2}t\right)$. Thus, the population remains finite on every finite time interval.

Theorem 4 **Local epidemic stability**

Consider a positive disease-free demographic equilibrium satisfying Equation 4. In the epidemiologically infected directions, the disease-free state is locally attracting if $R_{0}<1$ and unstable if $R_{0}>1$.

Proof. Linearization of the infected subsystem at the disease-free state gives $\frac{dz}{dt}=(F-V)z$. The next-generation matrix is $K=FV^{-1}$. The matrix F−V is stable in the infected directions precisely when ρ(K)<1. Therefore, $R_{0}<1$ implies that sufficiently small epidemiological perturbations decay to zero, whereas $R_{0}>1$ implies the existence of an unstable infected direction. Because the demographic part of the present model is homogeneous in population size, a positive disease-free demographic equilibrium satisfying Equation 4 is not isolated but belongs to a family of equilibria. Hence, the theorem concerns stability with respect to epidemiological perturbations rather than asymptotic convergence to one particular absolute population size.

A disease-free state is characterized by the absence of exposed and infectious individuals $E_{g,i}^{*}=I_{g,i}^{a,*}=I_{g,i}^{s,*}=0$, together with $E_{g,a}^{\;f,*}=I_{g,a}^{a,f,*}=I_{g,a}^{s,f,*}=0$. For the baseline disease-free state, we additionally assume that no infection-induced physiological fertility impairment is initially present. Consequently, the remaining population occupies the susceptible demographic classes. Let $b^{*}\;:\;=\mathrm{births}(x^{*})$. At a stationary disease-free demographic state, the susceptible populations satisfy

$$0=\tau_{g}b^{*}-(d_{g,c}+\alpha_{ca})S_{g,c}^{*},$$

$$0=\alpha_{ca}S_{g,c}^{*}-(d_{g,a}+\alpha_{ae})S_{g,a}^{*},$$

and

$$0=\alpha_{ae}S_{g,a}^{*}-(d_{g,e}+\alpha_{ed})S_{g,e}^{*}.$$

Hence,

$$S_{g,c}^{*}=\frac{\tau_{g}}{d_{g,c}+\alpha_{ca}}b^{*},\;S_{g,a}^{*}=C_{g}b^{*},\;C_{g}\;:\;=\frac{\tau_{g}\alpha_{ca}}{\left(d_{g,c}+\alpha_{ca})(d_{g,a}+\alpha_{ae}\right)},\;S_{g,e}^{*}=\frac{\alpha_{ae}}{d_{g,e}+\alpha_{ed}}C_{g}b^{*}.$$

At the disease-free state, all adults are in the normal-fertility class, so Equation 3 reduces to $b^{*}=\omega \min(S_{m,a}^{*},S_{\;f,a}^{*})$. Substitution of $S_{g,a}^{*}=C_{g}b^{*}$ gives $b^{*}=\omega \min(C_{m},C_{f})b^{*}$. Therefore, either $b^{*}=0$, which corresponds to the trivial zero-population equilibrium, or a positive stationary demographic state must satisfy the demographic replacement condition

ωmin(Cm,Cf)=1. (4)

Moving to the Lyapunov condition for global disease elimination. Let $\bar{S}$ be a disease-free demographic upper bound such that the susceptible components satisfy $S(t)\leq \bar{S}$ componentwise throughout the epidemiologically feasible region under consideration. Let us define $\bar{F}=F(\bar{S})$ and suppose that $\rho (\bar{F}V^{-1})<1$. Then $A=\bar{F}-V$ is a Hurwitz Metzler matrix. Consequently, there exists a vector q≫0 such that $q^{T}A\ll 0$. Consider the linear Lyapunov function

$$L(z)=q^{T}z.$$

Clearly L(z)>0 for z≠0 and L(0)=0. Using the upper bound on the susceptible population gives $\dot{z}\leq Az$, and therefore $\dot{L}=q^{T}\dot{z}\leq q^{T}Az<0$ for z≠0. It follows that z(t)⟶0 as t⟶∞. Thus, under the sufficient threshold condition $\rho (\bar{F}V^{-1})<1$, the infection-free epidemiological state is globally attracting within the corresponding feasible demographic region. This global result concerns elimination of the infected compartments. We do not claim global asymptotic stability of one particular positive equilibrium of the complete demographic–epidemiological system because, as shown above, the current birth and demographic structure does not generally generate an isolated positive demographic equilibrium.

Finally, we characterize the endemic states. Namely, for $R_{0}>1$, the disease-free epidemiological state becomes unstable and persistent transmission may occur. An endemic equilibrium, when it exists, is a non-negative stationary solution $x_{\mathrm{end}}^{*}$ satisfying $F(x_{\mathrm{end}}^{*})=0$ and

$$\sum_{g\in \{m,f\}}\left[\sum_{i\in \{c,a,e\}}\left(I_{g,i}^{a,*}+I_{g,i}^{s,*}\right)+I_{g,a}^{a,f,*}+I_{g,a}^{s,f,*}\right]>0.$$

Because the complete model simultaneously couples epidemiological progression, age transitions, immunity loss, mortality, infection-induced fertility impairment, and the nonlinear two-gender birth function, a useful closed-form expression for the endemic state is not generally available. Parameter-specific endemic states can instead be obtained by solving the stationary nonlinear system numerically, and their local stability can be determined from the eigenvalues of the corresponding Jacobian matrix.

### 3.2 Numerical solution

The nonlinear system in Equation 2 does not admit a useful closed-form analytical solution for the parameterized scenarios considered in this study. We therefore solve the coupled system numerically. Let x(t) denote the complete vector of epidemiological, demographic, and fertility-related state variables and write the model compactly as

$$\frac{dx}{dt}=F(x).$$

A first-order forward Euler discretization gives

$$x_{n+1}=x_{n}+\Delta t\;F(x_{n}),$$

where $t_{n}=n\Delta t$. All transition terms at iteration n, including infection, epidemiological progression, recovery, loss of immunity, mortality, aging, fertility-status transitions, and births, are evaluated from the same state vector $x_{n}$, and the resulting compartments are updated synchronously. The force of infection at time step n is first computed from the complete infectious population. New exposed individuals are then obtained by applying this force of infection to each susceptible demographic compartment. The remaining epidemiological transitions follow Equation 2: exposed individuals progress to the asymptomatic or symptomatic infectious states according to ρ and 1−ρ, respectively; infectious individuals subsequently recover or leave the population through disease-associated mortality; recovered individuals lose immunity at rate δ; and symptomatic adults who recover enter the physiological fertility-impaired class according to the impairment probability π. Demographic transitions due to aging and background mortality are applied in the same numerical step. Finally, the number of births is evaluated from Equation 3 using the adult male and female populations and their corresponding fertility states. For a simulation horizon of

$$T_{\mathrm{final}}=3,650\;\text{days},$$

we use

Δt=INSERTNUMERICALSTEPSIZEdays,

corresponding to

$$N_{T}=\frac{3,650}{\Delta t}$$

numerical integration steps. The time step is selected sufficiently small to preserve non-negativity of the numerical trajectories and to resolve the fastest epidemiological transition rate in the model. Numerical stability is additionally checked by repeating representative simulations with Δt/2 and verifying that the resulting population-level fertility trajectories are effectively unchanged. The control and pandemic scenarios are solved using the same initial demographic configuration and numerical procedure. In the control scenario, transmission is disabled by setting

β=0.

For experiments in which model parameters are sampled from the ranges reported in Table 2, the differential-equation solution is deterministic conditional on a particular sampled parameter vector. Repeated simulations therefore quantify variation induced by the sampled epidemiological and demographic parameters. The number of independent parameter realizations used for each experiment is reported together with the corresponding figure or table.

## 4 In silico investigation

In this section, we explore the TFR dynamics in a population using the proposed model. First, we define the TFR metric which is used to study the relationship between the pandemic spread and TFR. Afterward, we outline the experimental setup using the COVID-19 pandemic and cities from both Europe and America. Finally, we present the results obtained.

### 4.1 Evaluation metric

In an effort to capture the effect of the pandemic spread on TFR over time, we define a relative metric that computes the change that occurred due to a pandemic to the same configuration without a pandemic. Hence, we first computed the average number of individuals born in a timeframe $[t_{0},t_{f}],t_{0}<t_{f}$ such that the pandemic is not present where $t_{0}$ and $t_{f}$ are the beginning and end of the timeframe, respectively. Formally, one can use the same model with β=0. This “control” quanta is denoted by $B_{\mathrm{control}}$ and formally defined by $\left(\sum_{t=t_{0}}^{t_{f}}\mathrm{born}(t)\right)/(t_{f}-t_{0})$. Intuitively, $B_{\mathrm{control}}$ is the TFR (96–99). Similarly, for a given pandemic, the “case” quanta is denoted by $B_{\mathrm{case}}$ and computed identically. Based on these values, the average TFR due to the pandemic, B, takes the form:

$$B\;:\;=\frac{B_{\mathrm{control}}-B_{\mathrm{case}}}{B_{\mathrm{control}}}.$$

### 4.2 Experimental setup

For the purpose of capturing real-world configuration for the proposed model, we adopted the COVID-19 pandemic for the pandemic dynamic and used the demographic distribution of 10 central cities around the world. Table 2 presents the parameters and their values as these are used by the proposed model. In a complementary manner, Table 1 summarizes the population size, age distribution, and gender distribution for 10 real cities, from all over the world.

**Table 1:** The model’s initial condition for 10 real-world cities.

City	Size	TFR	Age (%)			Gender (%)		Source
			Children	Adult	Elderly	Female	Male	
Delhi, India	16.78 M	2.100	37.2	43.4	19.4	46.5	53.5	Ministry of Home Affairs, 2011
Shanghai, China	24.87 M	1.281	12.6	35.3	52.1	48.2	51.8	2020, China National Bureau of Statistics, Shanghai Municipal Bureau of Statistics
Paris, France	2.14 M	1.460	16.2	49.4	34.4	53.0	47.0	2020 Institut National de la Statistique et des Études Économiques, France
Istanbul, Turkey	15.46 M	2.046	25.6	45.0	29.4	49.9	50.1	2020 UrbiStat
London, UK	8.78 M	1.530	21.5	47.5	31.0	51.5	48.5	UK Office for National Statistics (2021)
Toronto, Canada	2.79 M	1.440	16.1	40.9	43.0	51.6	48.4	2021 Toronto Census
Tel Aviv, Israel	0.46 M	3.000	18.4	38.7	42.9	50.3	49.7	2021 Central Bureau of Statistics, The State of Israel
New York, USA	19.99 M	1.560	23.3	33.7	43.0	51.1	48.9	2022 American Community Survey 5-Year Estimates (https://data.census.gov/)
Sao Paulo, Brazil	11.45 M	1.630	45.6	37.4	17.0	53.0	47.0	2022 Instituto Brasileiro de Geografia e Estatistica
Berlin, Germany	3.80 M	1.616	17.0	39.0	44.0	51.0	49.0	Berlin-Brandenburg Statistics Office, 2024

**Table 2:** The model’s epidemiological parameters’ description, value ranges, and sources.

Symbol	Description	Value range	Source
T	Total number of simulation steps (1)	3,650	Assumed
Δt	Time steps in each simulation iteration (1)	1 day	Assumed
β	Average infection rate in days ($t^{-1}$)	$3.36\cdot 10^{-2}$–$1.73\cdot 10^{-1}$	(101)
ϕ	Exposed to infected rate in days ($t^{-1}$)	1/7–1/4	(101)
d	Non-disease related death rate, not included old-age, in days ($t^{-1}$)	$c-5.9\cdot 10^{-6},a-1.51\cdot 10^{-6},e-1.7\cdot 10^{-4}$	Sweden Government
ρ	Probability that an exposed individual develops an asymptomatic infection (1)	0.01–0.1	(101)
$\gamma_{s}$	Symptomatically infected individual recovery rate ($t^{-1}$)	0.90–1.00	(101)
$\gamma_{a}$	Asymptomatically infected individual recovery rate ($t^{-1}$)	0.98–1.00	(101)
λ	Recovery rate from the disease (1)	0.96	(101)
δ	Immunity decay rate ($t^{-1}$)	$1.1\cdot 10^{-2}$	(102)
$\alpha_{ca}$	Child to adult growth rate in days ($t^{-1}$)	$1.52\cdot 10^{-4}$	(100)
$\alpha_{ae}$	Adult to elderly growth rate in days ($t^{-1}$)	$1.10\cdot 10^{-4}$	(100)
$\alpha_{ed}$	Elderly to death due to age rate in days ($t^{-1}$)	$7.94\cdot 10^{-5}$	(100)
ω	Born rate in days ($t^{-1}$)	$1.55\cdot 10^{-4}$–$1.89\cdot 10^{-4}$	(103)
π	Probability that a symptomatic recovered adult develops physiological fertility impairment (1)	Scenario-dependent	Assumed
r	Proportional fertility reduction among physiologically impaired adults (1)	0.04–0.06	(104)
τ	Probability for a newborn is a male (1)	0.485–0.515	Assumed

For simplicity, we classify individuals aged 0 to 17 as children, 18 to 43 as adults, and 44 and older as elderly. This classification is performed with respect to the age range which is responsible for 95% of the births, in a symmetric manner (100). For the life expectancy, which is used to calculate the rate of death due to age ($\alpha_{ed}$), we used the one reported for the USA at 2024 – 78.5 years.^1^ For the non-disease or age-related death rate, we used the average rate for each age-group, as defined above based on the data from the Sweden Government.^2^ Moreover, empirically, human sex ratios at birth are close to half with modest cross-population and temporal variation; we therefore set a baseline τ=0.50 and explore τ∈[0.485,0.515] to cover typical demographic variation while allowing sensitivity headroom. As a benchmark, short-run pathogen shocks like COVID-19 produced single-digit TFR: U.S. births fell about 4% in 2020 overall, and cross-country monthly data show around 4%–6% year-over-year drops at the late-2020 trough; we therefore adopt 4%–6% as a conservative, empirically grounded range for the peak-period decline.

Notably, we set $\beta ,\phi ,\rho ,\gamma^{s},\gamma^{a},\lambda ,\delta$ identical across age and sex (and symptom/fertility status where applicable), using the baseline values in Table 2 due to lack of group-level data. For the sensitivity analysis, the default value is set to be the middle value of each range.

In addition, to simulate realistic scenarios, we used the socio-demographic distribution of 10 large cities from all over the world in terms of age distribution and gender distribution. Table 1 summarizes these configurations.

We implemented the proposed model (Equation 2) using an Agent-Based Simulation (ABS) approach, following the methodology outlined by (91). Formally, consider a population of agents, A, such that each agent in the population a∈A is defined by a timed finite state machine (105) as follows: $a\;:\;=(p_{a},p_{g},p_{e},p_{f}$ where $p_{a}\in \{c,a,e\}$ is the agent’s age, $p_{g}\in \{m,f\}$ is the agent’s gender, $p_{e}\in \{S,E,I^{s},I^{a},R\}$ is the agent’s epidemiological state, and $p_{f}\in \{n,f\}$ is the fertility state of the individual (no-issues, and fertility-issues). These agents interact in discrete, finite time steps t∈[1,…,T] such that T<∞. The ABS approach requires defining agents and their three types of interactions: agent-agent, agent-environment, and spontaneous (i.e., interactions that depend solely on the agent’s state and time) (106). The simulation begins by generating an agent population. Then, iteratively, until a predefined number of time steps is reached (T), the simulation updates the dynamics by solving Equation 2. Notably, fertility impairment is treated as an absorbing physiological status during adulthood: once an adult enters the fertility-impaired compartment, the individual remains fertility-impaired until aging into the elderly group or leaving the population. This assumption reflects the focus of the present study on long-term physiological fertility reduction following symptomatic infection. For more technical details, we refer the reader to (91).

### 4.3 Results

In this section, we present the obtained results of the simulation. Initially, we explore the change of the fertility rate (B) over time due to a pandemic. Figure 2 illustrates the change in the fertility rate B over time for the cities listed in Table 1 during two types of pandemics—(a) without a physiological fertility decline (ξ=0) and (b) with a physiological fertility decline (ξ=0.05). The results are shown as the mean of n=100 simulation repetitions for each city. One can notice that, unsurprisingly, for the case where there is a physiological fertility decline due to the pandemic (ξ>0), the decline in the average fertility rate is higher. The differences between the cities dynamics also increase as can be observed by the value of B at T=3650. In addition, the decline is increasing up to some point for all cities. Most cities exhibit a late-phase plateau in B; others (e.g., Sao Paulo) still trend upward over the plotted horizon which can be associated with the end of the pandemic or at least its significant part.

**Figure 2:**
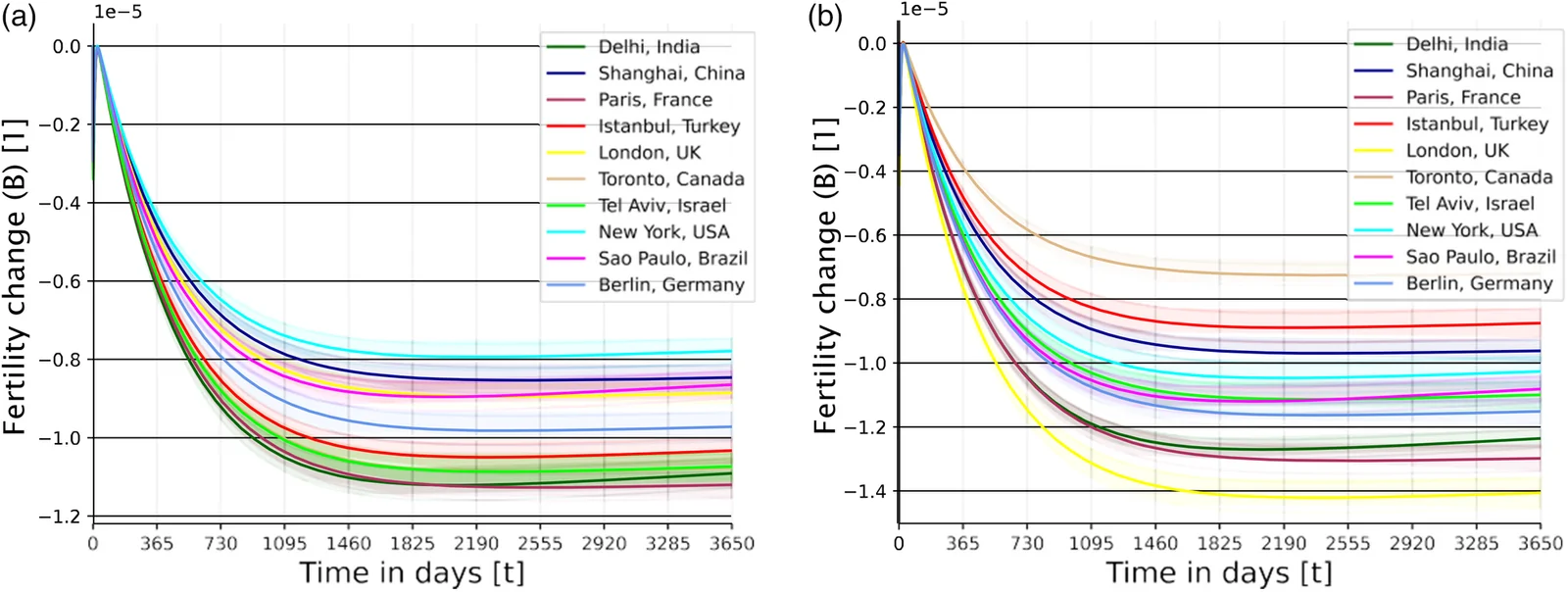
Fertility rate dynamics, B, over time, for the cities in Table 1, divided into **(a)** a pandemic but without physiological fertility decline and **(b)** a pandemic with physiological fertility decline. The results are shown as the mean of n=100 simulation repetitions. **(a)** With the pandemic, without physiological fertility decline (ξ=0). **(b)** With the pandemic, with physiological fertility decline (r=0.05, κ=0.95).

From these results, it is clear that pandemics cause a decline in TFR, as B increases above zero and approaches a long-term plateau in most cities. Thus, larger values of B indicate a stronger pandemic-related fertility decline. Thus, we discuss the effect of the pandemic on the physiological fertility decline (|B|) to align with the intuitive direction of the larger |B| value associated with a higher decline.

As not all pandemics are identical (1), we next explore the sensitivity of three main parameters on the decline in fertility rate—the average infection rate (β), probability a newborn is male (τ), immunity decay rate (δ), andthe proportional fertility reduction among impaired adults (r). Figure 3 presents one-dimensional sensitivity of the TFR as a function of these parameters, such that the results are shown as the mean ± standard deviation of n=100 simulation repetitions, 10 for each city in Table 1. Starting with the average infection rate (β) (Figure 3a), one can observe a monotonic increase in the fertility rate with respect to β, which itself increases as β increases. Notably, the entropy of the system also increases, as indicated by the increase in the standard deviation. Next, the TFR with respect to the immunity decay rate (δ) also increases, on average, when δ increases while not monotonically. Remarkably, the entropy first decreases with δ of value 0.009 and increases again for δ values larger than 0.011. Finally, the decline in fertility is increasing linearly [with a coefficient of determination of $R^{2}=0.97$ obtained from a linear regression model fit (107)] with respect to the drop in fertility due to the pathogen (ξ) with also slow increase in the entropy as the value of ξ increase.

**Figure 3:**
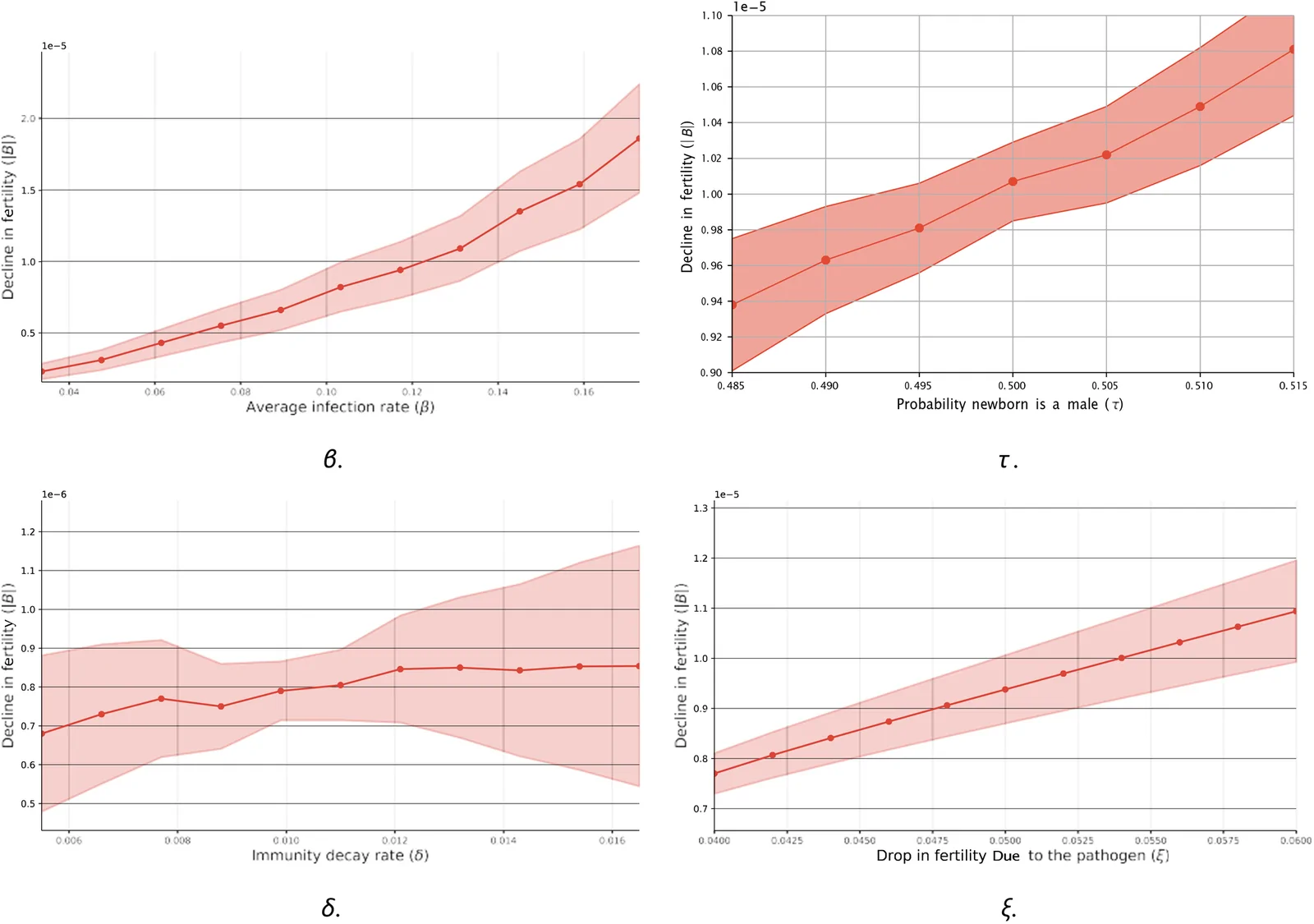
One-dimensional sensitivity of the TFR as a function of the average infection rate (β), probability a newborn is male (τ), immunity decay rate (δ), and the drop in fertility rate due to the pathogen (ξ). The results are shown as the mean ± standard deviation of n=100 simulation repetitions, 10 for each city in Table 1.

Furthermore, we explored the joint effect of the demographic and pandemic properties on the TFR. To this end, Figure 4 shows a two-dimensional analysis of the TFR with respect to three pairs of model parameters: ξ×ω, ϕ×β, and $\gamma_{s}\times \beta$ such that the results are shown as the mean of the 10 cities from Table 1 with n=10 simulation repetitions for each city. We used n=10 repetitions for the 2D heatmaps due to computational cost; the qualitative rankings match the n=100 of the 1D analyses. Focusing on Figure 4a, one can notice a monotonic and linear increase in |B| with respect to ξ while not much change to the value of ω. From Figure 4b, one can infer that the relationship between |B| and both ϕ and β is highly non-linear. Finally, Figure 4c presents a more ordered relationship where a joint increase in both $\gamma_{s}$ and β results in a smaller |B| compared to the case where $\gamma_{s}$ is small and β and the mirrored case.

**Figure 4:**
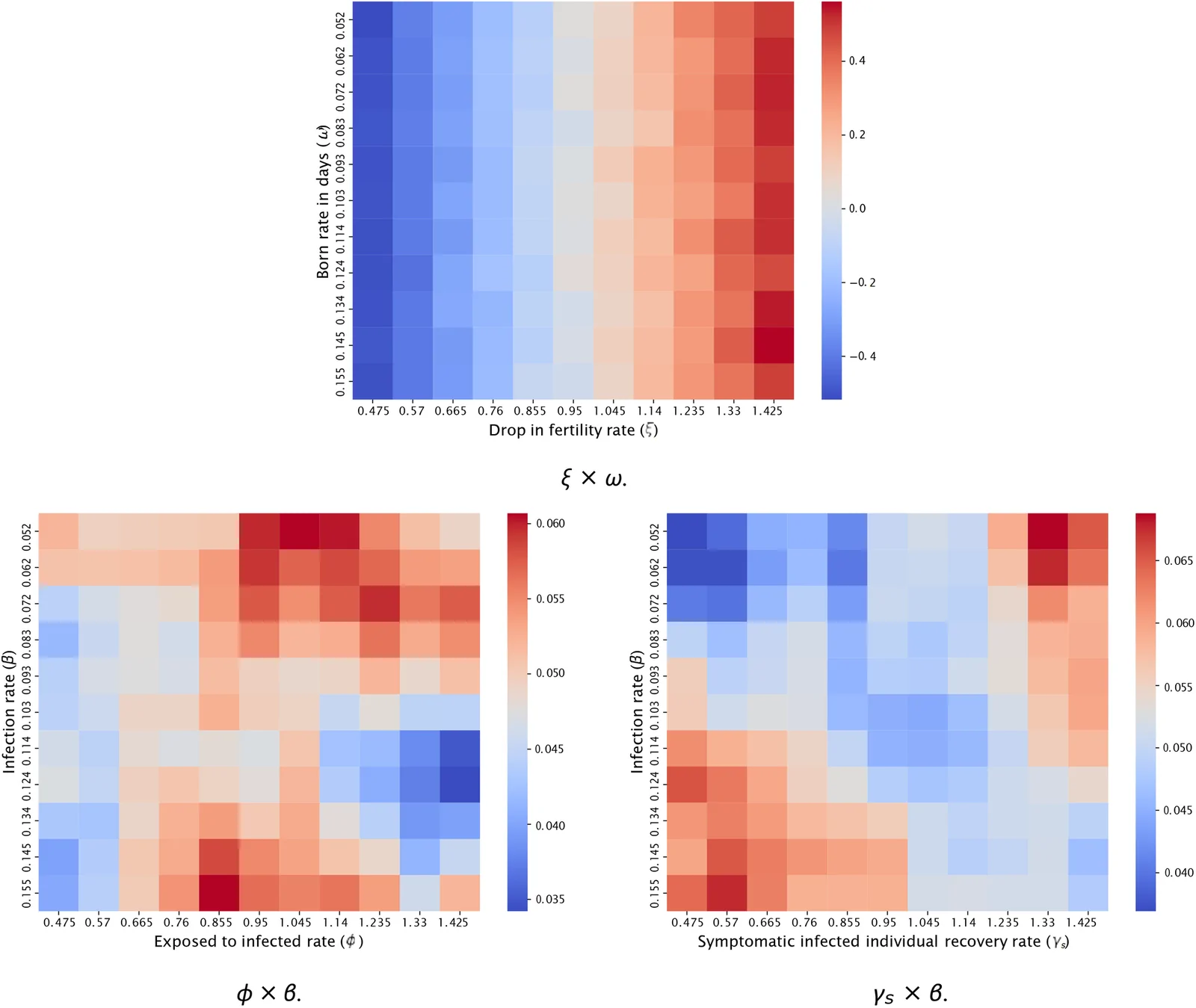
Two dimensional analysis of the TFR with respect to pandemic properties. The results are shown as the mean of the 10 cities from Table 1 with n=10 simulation repetitions for each city.

Extending this analysis, we computed the one-dimensional sensitivity analysis (108) of each of the epidemiological parameters on the decline in fertility. Table 3 outlines the results of the analysis. presenting mean change obtained from a linear regression (109) model fitted on n=100 simulations ranging between 50% and 150% with 1% step size from the original value presented in Table 2. This model averages results across the cities mentioned in Table 1 with 10 samples from each city. We obtained a coefficient of determination of $R^{2}=0.803$ indicating the values of the linear regression fairly capture the sensitivity of the decline in fertility respectively to each of the model’s parameters. The sensitivity analysis indicates that only a subset of the model parameters are statistically significant predictors of fertility decline. In particular, β, $\gamma_{s}$, λ, δ, and $\alpha_{ed}$ are significant at the 5% level, while several demographic transition and background mortality parameters are not statistically significant. The fertility-related parameter is also significant, but the table should be interpreted with caution because the relationship between fertility impairment and population-level TFR may be nonlinear.

**Table 3:** Sensitivity analysis of the epidemiological parameters.

Parameter	Value	*p*-value	Confidence interval
β	$1.55\cdot 10^{-1}$	$8.80\cdot 10^{-16}$	$\left(7.94\cdot 10^{5},8.20\cdot 10^{-5}\right)$
ϕ	$2.73\cdot 10^{-1}$	$5.94\cdot 10^{-1}$	$\left(-1.89\cdot 10^{-6},3.38\cdot 10^{-6}\right)$
$d_{c}$	$8.85\cdot 10^{-6}$	$3.37\cdot 10^{-1}$	$\left(-3.20\cdot 10^{-2},1.01\cdot 10^{-1}\right)$
$d_{a}$	$2.27\cdot 10^{-6}$	$3.64\cdot 10^{-1}$	$\left(-2.34\cdot 10^{-1},6.81\cdot 10^{-1}\right)$
$d_{e}$	$2.55\cdot 10^{-4}$	$9.47\cdot 10^{-1}$	$\left(-4.72\cdot 10^{-3},4.40\cdot 10^{-3}\right)$
ρ	$8.25\cdot 10^{-2}$	$7.93\cdot 10^{-1}$	$\left(-9.04\cdot 10^{-6},1.19\cdot 10^{-5}\right)$
$\gamma_{s}$	$1.43\cdot 10^{0}$	$2.45\cdot 10^{-6}$	$\left(-1.27\cdot 10^{-5},-8.67\cdot 10^{-6}\right)$
$\gamma_{a}$	$1.49\cdot 10^{0}$	$2.59\cdot 10^{-1}$	$\left(-1.15\cdot 10^{-6},2.75\cdot 10^{-7}\right)$
λ	$1.44\cdot 10^{0}$	$1.03\cdot 10^{-14}$	$\left(1.80\cdot 10^{-5},1.88\cdot 10^{-5}\right)$
δ	$1.65\cdot 10^{-2}$	$9.30\cdot 10^{-4}$	$\left(8.37\cdot 10^{-5},1.98\cdot 10^{-4}\right)$
$\alpha_{ca}$	$2.28\cdot 10^{-4}$	$3.62\cdot 10^{-1}$	$\left(-8.04\cdot 10^{-3},2.75\cdot 10^{-3}\right)$
$\alpha_{ae}$	$1.65\cdot 10^{-4}$	$6.29\cdot 10^{-1}$	$\left(-7.76\cdot 10^{-3},4.61\cdot 10^{-3}\right)$
$\alpha_{ed}$	$1.19\cdot 10^{-4}$	$6.47\cdot 10^{-3}$	$\left(3.57\cdot 10^{-3},1.25\cdot 10^{-2}\right)$
ω	$2.58\cdot 10^{-4}$	$2.70\cdot 10^{-1}$	$\left(-1.24\cdot 10^{-3},4.96\cdot 10^{-3}\right)$
ξ	$7.50\cdot 10^{-2}$	$1.38\cdot 10^{-2}$	$\left(-2.48\cdot 10^{-5},-5.39\cdot 10^{-6}\right)$
τ	$7.50\cdot 10^{-1}$	$6.08\cdot 10^{-1}$	$\left(-1.33\cdot 10^{-6},2.31\cdot 10^{-6}\right)$
ξ	$1.43\cdot 10^{0}$	$7.30\cdot 10^{-12}$	$\left(1.84\cdot 10^{-5},2.01\cdot 10^{-5}\right)$

The table provides parameter values, *p*-values, and 95% confidence intervals from a linear regression model.

## 5 Discussion

In this study, we proposed a novel model that integrates the pandemic spread model based on the popular SIR model with several socio-demographic, including genders, age groups, and, most importantly—the impact of infectious with the pandemic’s pathogen on fertility due to physiological issues. In order to evaluate the effect of a pandemic on a population’s fertility over time, we *in silico* explored a COVID-19-like pathogen spread with a more aggressive fertility effect on 10 large-size cities from around the world.

Initially, we compare two scenarios of a pandemic, where one does not have a physiological fertility decline for infected individuals and one that does. As expected, the latter resulted in a higher overall fertility decline in the population, as demonstrated in Figure 2a (110). However, the introduction of such physiological fertility decline has a complex and non-linear effect on the pandemic spread over long periods of time (of several years or more), which can alter significantly the overall physiological fertility decline as illustrated by Figure 2b. In particular, focusing on New York (USA), its change in TFR was more significantly impacted compared to Istanbul (Turkey).

These results were obtained in the context of a COVID-19-like pandemic and recognizing that pandemics can vary significantly in their characteristics, such as average infection rate, immunity decay rate, and the extent of TFR induced by the pathogen, it is crucial to assess the broader implications of these variations (111, 112). To this end, we conducted a comprehensive sensitivity analysis, systematically examining the effects of these three key parameters on population-level TFR, as presented in Figure 3. Specifically, we find a non-linear and monotonic increase in the decline in fertility with the infection rate (β) (113). This outcome can be associated with the increased amount of individuals infected and, therefore, die or their physiological fertility decline. Notably, the standard deviation is also increased as the infection rate (β) indicates the system is becoming more chaotic. Similarly, when focusing on the decline in fertility due to the pathogen (ξ), almost the same pattern emerges, but this time linear rather than non-linear. Distinctly, an increase in the average immunity decay (δ) has a minor effect on the decline in fertility with non-monotonic and slight increase as δ increases.

Furthermore, when considering the dynamic’s sensitivity for two parameters at the same time, Figure 4 shows Panel (a) illustrates the relationship between the drop in fertility rate (ξ) and birth rate bands (ω), revealing a clear gradient where higher physiological fertility decline is associated with lower birth rates, suggesting that pandemics disproportionately affect regions with already declining fertility trends, following the same trend as in Figure 3d and also agreeing with previous studies (114–116). Panel (b) presents the interplay between the exposed-to-infected transition rate (ϕ) and infection rate (β), revealing highly non-linear behavior where moderate ϕ with large and small β associated with a higher decline in fertility while other cases, such as high ϕ with beta∼0.124 resulting in a lower decline in fertility. Finally, panel (c) depicts the relationship between the symptomatic infected recovery rate ($\gamma_{i}$) and infection rate (β), indicating that higher recovery rates mitigate the effects of increasing infection rates on TFR, agreeing with previous studies (117, 118).

Extending this analysis to all the model’s parameters simultaneously, Table 3 reveals a linear correlation between the TFR and all the parameters such that the recovery rate from the disease (λ) is the most significant one followed by the decline in fertility due to the pathogen (ξ) and the average infection rate (β), all in a statistically significant manner. These outcomes imply that policies aimed at improving healthcare infrastructure, accelerating treatment, and reducing disease duration could mitigate TFR more effectively than interventions targeting infection rates alone. This underscores the critical role of post-infection recovery in demographic stability.

This study is not without limitations. First, our model assumes homogeneous populations with respect to fertility impacts and does not account for potential variations across different socio-economic or ethnic groups (119, 120). Future work should aim to incorporate these demographic variances to enhance the model’s accuracy and applicability. Second, our current approach focuses on a single pathogen and its immediate aftermath. Given the potential for multiple waves of infection or the simultaneous occurrence of different pathogens, future models should consider more complex scenarios involving co-infections or successive pandemics, integrating multi-pathogen models (121, 122). Third, pandemics often lead to economic downturns, which can have profound effects on fertility rates. Namely, Economic hardships, such as job losses, reduced income, and increased financial uncertainty, can lead to delayed or reduced childbearing (123, 124). Future research can integrate economic data into the model to provide a more comprehensive understanding of the interplay between economic conditions and fertility trends during health crises. Finally, the psychological impact of pandemics on fertility decisions, such as changes in personal aspirations and risk perceptions, remains underexplored (38, 125). Integrating psychological and behavioral data into the model could provide a more holistic understanding of fertility dynamics during and after pandemics.

Based on recent research by Aassve et al. (110, 126), which highlights how pandemic effects on fertility operate through multiple physiological and socioeconomic pathways, we plan to propose a mathematical model that captures these complex dynamics. The model will integrate direct physiological impacts on reproductive health, socioeconomic disruptions to family planning, and the resulting demographic ripple effects that persist beyond the immediate crisis. Through ODE and ABS incorporating age-structured populations, gender-specific effects, and both immediate and long-term fertility impacts, we aim to quantitatively demonstrate how these interacting mechanisms shape population dynamics during and after pandemic events.

Taken jointly, our results show that even a moderate impact of the pandemic on fertility can lead to a significant decline in birth rates over time, especially in cities with low birth rates to begin with. As such, policymakers should take the pandemic’s effect on fertility due to physiologically related reasons into account when preparing social, economic, and demographic preparation plans for future pandemics.

## Data Availability

The original contributions presented in the study are included in the article/Supplementary Material, further inquiries can be directed to the corresponding author. The code of the project is publicly available in the project's Github repository code availability: https://github.com/teddy4445/pandemic_ fertility_model_solver.

## Footnotes

^1^ https://population.un.org/wpp/Download/Standard/MostUsed/

^2^ https://www.statistikdatabasen.scb.se/pxweb/en/ssd/START__BE__BE0101__BE0101I/Dodstal/
